# The epidemiology of varicella and effectiveness of varicella vaccine in Ganyu, China: a long-term community surveillance study

**DOI:** 10.1186/s12889-023-16304-4

**Published:** 2023-09-28

**Authors:** Lingxian Qiu, Sheng Liu, Minglei Zhang, Guohua Zhong, Siying Peng, Jiali Quan, Hongyan Lin, Xiaowen Hu, Kongxin Zhu, Xingcheng Huang, Junchao Peng, Yue Huang, Shoujie Huang, Ting Wu, Jinbo Xu, Zifang Dong, Qi Liang, Wei Wang, Yingying Su, Jun Zhang, Ningshao Xia

**Affiliations:** 1 State Key Laboratory of Vaccines for Infectious Diseases, Xiang An Biomedicine Laboratory, Department of Laboratory Medicine, School of Public Health, Xiamen University, Xiamen, Fujian China; 2 National Institute of Diagnostics and Vaccine Development in Infectious Diseases, State Key Laboratory of Molecular Vaccinology and Molecular Diagnostics, Collaborative Innovation Center of Biologic Products, National Innovation Platform for Industry-Education Integration in Vaccine Research, NMPA Key Laboratory for Research and Evaluation of Infectious Disease Diagnostic Technology, the Research Aff of Frontier Technology of Structural Vaccinology of Chinese Academy of Medical Sciences, Xiamen University, Xiamen, Fujian China; 3 Ganyu County Center for Disease Control and Prevention, Ganyu County, Lianyungang, Jiangsu China; 4 Information Technology and Laboratory Management Center, Wuyi University, Wuyishan, Fujian China; 5 Jiangsu Provincial Center for Disease Control and Prevention, Nanjing, Jiangsu China; 6 The Research Aff of Frontier Technology of Structural Vaccinology of Chinese Academy of Medical Sciences, Xiamen, China

**Keywords:** Community surveillance, Epidemiology, Long-term, Varicella, Vaccine effectiveness

## Abstract

**Background:**

The real-world data of long-term protection under moderate vaccination coverage is limited. This study aimed to evaluate varicella epidemiology and the long-term effectiveness under moderate coverage levels in Ganyu District, Lianyungang City, Jiangsu Province.

**Methods:**

This was a population-based, retrospective birth cohort study based on the immunization information system (IIS) and the National Notifiable Disease Surveillance System (NNDSS) in Ganyu District. Varicella cases reported from 2009 to 2020 were included to describe the epidemiology of varicella, and eleven-year consecutive birth cohorts (2008–2018) were included to estimate the vaccine effectiveness (VE) of varicella by Cox regression analysis.

**Results:**

A total of 155,232 native children and 3,251 varicella cases were included. The vaccination coverage was moderate with 37.1%, correspondingly, the annual incidence of varicella infection increased 4.4-fold from 2009 to 2020. A shift of the varicella cases to older age groups was observed, with the peak proportion of cases shifting from 5–6 year-old to 7–8 year-old. The adjusted effectiveness of one dose of vaccine waned over time, and the adjusted VE decreased from 72.9% to 41.8% in the one-dose group.

**Conclusions:**

The insufficient vaccination coverage (37.1%) may have contributed in part to the rising annual incidence of varicella infection, and a shift of varicella cases to older age groups occurred. The effectiveness of one dose of varicella vaccine was moderate and waned over time. It is urgent to increase varicella vaccine coverage to 80% to reduce the incidence of varicella and prevent any potential shift in the age at infection in China.

## Introduction

Varicella (chickenpox) is a highly infectious disease predominant in childhood. Varicella is generally a self-limiting, mild disease in childhood but is more severe in adults, and severe complications can occur [1]. The World Health Organization (WHO) estimated that varicella disease causes 4.2 million severe complications leading to hospitalization and 4200 deaths worldwide annually [2]. The live attenuated varicella vaccine (Oka strain) was developed in 1974. In countries with routine universal varicella vaccination, significant reductions were observed in the morbidity, mortality, and health care costs associated with varicella-related disease [3–5].

Live-attenuated varicella vaccines were introduced in China in 1997 and have been recommended for children 12 months through 12 years old with a single-dose regimen [6]. However, the national strategy for varicella vaccination in China is to encourage people to receive the vaccine through the principle of voluntarism, and a meta-analysis showed that the overall one-dose varicella vaccine coverage was 61.1%, ranging from 20.8% to 97.8% [7]. With moderate vaccination coverage, the incidence of varicella increased from 3.17 per 100,000 in 2005 to 70.14 per 100,000 in 2019 [8, 9].

The effectiveness of the two-dose varicella vaccine can provide higher protection against varicella infections than the one-dose varicella vaccine (92% vs. 81%) [10]. During the era of the two-dose varicella vaccine implementation in the Unite State, the varicella incidence was lower in the two-dose era than that in the one-dose era (3.9 vs. 25.4/100,000 population) [11], and the breakthrough varicella incidence was also lower in the two-dose era (2.2% vs. 7.3%) [12]. Two doses of universal vaccination policies have been introduced in many countries, including the United States, Canada, Japan, Germany, and Luxembourg [1]. The two-dose schedule of varicella has been recommended since 2012 in some districts of China to further control the outbreaks of varicella [13].

However, the effectiveness data of one- and two-dose varicella vaccines were reported from areas with routine universal varicella vaccination or high vaccination coverage. Moreover, real-world data on long-term protection after vaccination are limited [1]. To fill this knowledge gap, this register-based birth cohort study aimed to describe varicella epidemiology and the one- and two-dose vaccine long-term effectiveness under moderate coverage levels in Ganyu District, Lianyungang City, Jiangsu Province.

## Materials and method

### Study design and study population

This was a population-based, retrospective birth cohort study based on the immunization information system (IIS) and the National Notifiable Disease Surveillance System (NNDSS) in Ganyu District, Lianyungang City, Jiangsu Province. The Ganyu District is situated in the northeastern region of Jiangsu Province within East China and the Yangtze River Delta area. As of 2019, the district had a population of one million residents. The average disposable income for urban residents was RMB 34,575 while that for their rural counterparts was RMB 19,100.

The IIS in Ganyu District is a population-based system, which contains basic information of children, individual vaccine immunization information, vaccine catch-up information, vaccine management information, adverse reaction information and cold chain information [14]. To estimate the effectiveness of the varicella vaccine, we selected eleven-year consecutive birth cohorts who were born between 2008 and 2018 and who were registered as permanent residents as the target population. Patients within the target population were excluded from the analysis when they met the following exclusion criteria: 1) children were diagnosed with varicella by doctors when the patients were aged less than 1-year-old; 2) children were vaccinated against varicella when they were aged less than 1-year-old; 3) children were vaccinated against varicella with more than two doses; 4) children were clinically diagnosed with varicella within 42 days after vaccination; 5) non-local or floating population; and 6) children who moved out of the area. The demographic and varicella vaccination information of the selected birth cohorts were obtained from the IIS.

In accordance with the WHO position paper, we categorized varicella vaccination coverage as high levels (above 70%), moderate levels (between 30 and 70%), and low levels (below 30%) [2].

The varicella cases were derived from the NNDSS. The NNDSS in China is the most fundamental and important infectious disease surveillance mechanism and is a passive reporting system covering all types of health facilities at all levels [15]. Since 2005, varicella cases have been reported to the NNDSS as other infectious disease in China. Since 1 July 2017, varicella surveillance has been mandated in Jiangsu Province, and it has been categorized as a Class “C” infectious disease [16]. This decision has increased the sensitivity of varicella monitoring in the province. Surveillance varicella case classification included probable cases, clinical cases, and laboratory-confirmed cases. A probable case is defined as a clinical manifestation with an acute onset of a diffuse maculopapulovesicular rash without another apparent cause. A varicella clinical case was defined as a case that met the probable case definition and was epidemiologically linked to other varicella or herpes zoster cases. A varicella laboratory confirmed case was defined as a case that met the probable case definition and in which the patient was laboratory confirmed with a positive serologic test for varicella-zoster immunoglobulin M (IgM) antibody, a fourfold or greater rise in serum varicella immunoglobulin G (IgG) antibody titer in two sera (2 to 4 weeks apart), isolation of varicella-zoster virus, or detection of varicella-zoster virus antigen by direct immunofluorescence antibody assay (DFA) or multiplex polymerase chain reaction (PCR) [8, 17]. In this study, the varicella clinical case and laboratory confirmed case information was collected from the NNDSS, and the data included name, date of birth, age, address, diagnosis(including date of onset and time of diagnosis), workplace or school, date of demise (if applicable), reporting physician, reporting time, reporting unit, updated records, review records, and deletion records.

#### Procedure

To estimate the effectiveness of the varicella vaccine, the exposure variable was varicella vaccination from January 1, 2009, to December 31, 2020, in the selected eleven-year consecutive birth cohorts who were born between 2008 and 2018, since children received varicella vaccination from age 1. The outcomes were varicella clinical case and laboratory confirmed cases, which were derived from the NNDSS and linked to the birth cohorts in the IIS by matching the names, date of birth, name of the parents or the address. Those patients for whom their vaccination records could not be verified were excluded from the analysis of vaccine effectiveness (VE). In the vaccinated cohorts, breakthrough varicella cases were considered outcomes and were defined as varicella cases occurring more than 42 days after vaccination [18]. In the unvaccinated cohorts, patients older than age 1 and with varicella were considered outcomes. To adjust for potential confounders, a set of covariates was formed, including age, year of birth, age at entry into cohort, and immunization management institution, which were obtained from the IIS.

#### Ethical approval and informed consent

The Medical Ethics Committee of Xiamen University reviewed and approved this protocol (approval no: XDYX202301K03). Data used in this study were obtained from NNDSS and IIS system, and all data were unidentified. A waiver of written informed consent was granted by the ethics committee.

#### Sample size calculation

All available data from NNDSS and IIS system that met inclusion criteria were collected and included in the analysis; no formal sample size calculation was performed.

#### Statistical analysis

The annual incidence of varicella was calculated as the number of varicella cases divided by the corresponding population estimates from the census data from Ganyu District. The calculation of annual incidence rates includes cases from all age groups. The change in the proportion of varicella cases by age was calculated in the 4-year Groups 2009–2011, 2012–2014, 2015–2017 and 2018–2020 to estimate whether there was a shift of varicella cases into the older age groups.

Vaccination coverage was determined as the proportion of children, in whom at least one dose of varicella vaccination was recorded, within the birth cohorts, which were based on specific ages. The cumulative incidence curves for the unvaccinated, one-dose and two-dose cohorts were estimated using the Kaplan‒Meier method, and the 95% CIs for each curve were estimated using the percentile bootstrap method. The follow-up time was calculated as the time from age 1 (for the unvaccinated cohort) or the date of the first vaccination (for the one-dose and two-dose cohorts) until the date of disease onset or the end of follow-up on December 31, 2020, whichever happened first. The crude incidence rate ratios (IRRs) were calculated by the incidence rates of the two groups as IRRs = incidence rate_vaccinated_/incidence rate_unvaccinated_. IRRs were estimated for one dose and two doses separately using the unvaccinated cohort as the reference group. The incidence rates were defined for each of the three groups as the number of varicella cases divided by the cumulative follow-up time in the group. The 95% confidence interval (95% CI) was calculated as (e^ln(uIRR)−1.96×SE^, e^ln(uIRR)+1.96×SE^). The standard error (SE) was defined as the open square of the sum of the inverses of the patients in the vaccinated and unvaccinated groups. In addition, the unadjusted hazard ratios (HRs) with 95% CIs were estimated using time-varying Cox regression analysis with varicella cases as the dependent variable, the immune status and follow-up time as a time-varying covariable. We included "unvaccinated" and "one-dose" as the time-varying variables; however, we excluded "two-dose" due to the small sample size. On this basis, adjusted HRs with 95% CI were also calculated, adjusting for birth year (2008–2018), age at cohort entry and immunization management institution (25 township health station of Ganyu county). VE was calculated as (1-HR) × 100.

All analyses were performed with SAS version 9.3 (SAS Inc., Cary, NC), and graphs were plotted using GraphPad Prism 8.0. Two-sided P values were reported with a significance level of *P* < 0.05.

## Result

### Study population

**Fig. 1:**
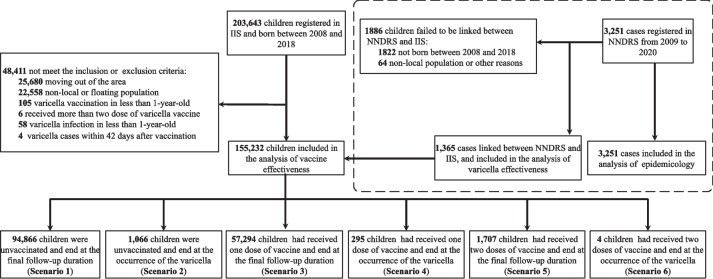
Study cohort enrollment process. The enrollment process of this study and the distribution of the enrollment population according to 6 scenarios with different vaccination backgrounds and outcomes

**Fig. 2:**
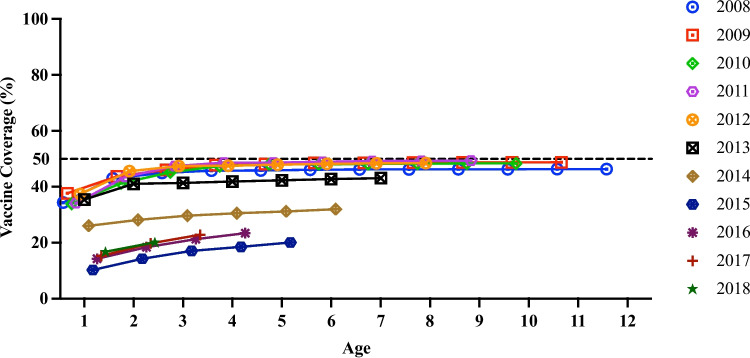
Vaccine coverage of varicella in each birth cohort from 2008 to 2018. *Vaccination rates are stratified by different birth cohorts, and based on the analysis group of varicella effectiveness

A total of 203,643 newborns in the IIS were screened for eligibility. A total of 48,411 were excluded, and 155,232 children were eligible, constituting the primary study cohort for the analysis of VE (Fig. 1). Overall, 95,932 children (61.8%) were not vaccinated, and 59,300 children were received at least one dose of varicella vaccine. Of these, 57,589 (37.1%) children received one dose of varicella vaccine at a mean age of 1.8 years (SD = 1.1), 1,711 (1.1%) children received two doses of varicella vaccine at a mean age of 5.5 years (SD = 2.1) and 95,932 children (61.8%) were not vaccinated during the follow-up period. (Table 1). For the 2008–2013 birth cohort, the vaccine coverage increased yearly from 1 to 3 years, then reached a plateau and remained at 43.1%-49.4%. The vaccine coverage of the 2014–2018 birth cohort was lower than that of the 2008–2013 birth cohort at the same age (Fig. 2) and reached 17.1%-29.7% at age 3.

**Table 1:** Baseline characteristics of children by varicella vaccination status in Ganyu district, Jiangsu Province (*N* = 155,232)^a^

Variables	Total	Follow-up time	Unvaccinated	1-dose	2-dose
		(Mean ± SD)	n (%)	n (%)	n (%)
Total population	155,232	-	95,932(61.8%)	57,589(37.1%)	1,711(1.1%)
No of cases	1,365	-	1,066(78.1%)	295(21.6%)	4(0.3%)
Average age at cohort entry					
Mean ± SD	1.3 ± 0.8	-	1.0 ± 0.0	1.8 ± 1.1	5.5 ± 2.1
Birth cohort					
2008	15,681	11.1 ± 0.9	8,416(53.7%)	7,225(46.1%)	40(0.3%)
2009	16,051	10.1 ± 1.0	8,225(51.2%)	7,773(48.4%)	53(0.3%)
2010	15,513	9.0 ± 1.1	8,015(51.7%)	7,372(47.5%)	126(0.8%)
2011	15,469	8.0 ± 1.0	7,837(50.7%)	7,438(48.1%)	194(1.3%)
2012	16,780	7.1 ± 0.8	8,648(51.5%)	7,716(46.0%)	416(2.5%)
2013	14,491	6.2 ± 0.8	8,253(57.0%)	5,900(40.7%)	338(2.3%)
2014	13,900	5.2 ± 0.8	9,459(68.1%)	4,176(30.0%)	265(1.9%)
2015	12,406	4.2 ± 0.9	9,912(79.9%)	2,357(19.0%)	137(1.1%)
2016	13,234	3.2 ± 0.7	10,136(76.6%)	2,997(22.6%)	101(0.8%)
2017	11,465	2.3 ± 0.6	8,850(77.2%)	2,579(22.5%)	36(0.3%)
2018	10,242	1.4 ± 0.4	8,181(79.9%)	2,056(20.1%)	5(0.0%)

^a^The result was based on the analysis group of varicella effectiveness

#### The annual incidence of varicella infection in Ganyu District

**Fig. 3:**
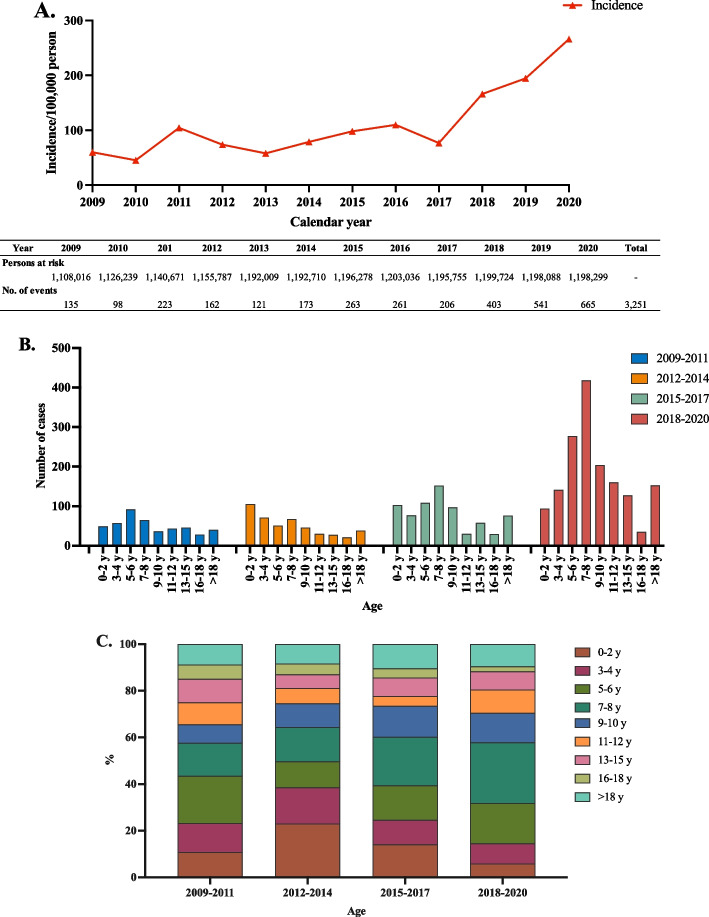
The epidemiological features and vaccine coverage of varicella. **A** The annual incidence of varicella from 2009 to 2020. **B** Age distribution of the number of varicella cases from 2009 to 2020. **C** The age distribution of the constituent ratio of varicella cases from 2009 to 2020. *The results were based on an epidemiological analysis

A total of 3,251 cases (99% were clinically confirmed cases) of varicella were reported in the NNDRS from January 1, 2009, to December 31, 2020, in Ganyu District (Fig. 1). The annual incidence of varicella infection increased 4.4-fold from 59.8/100,000 in 2009 to 265.9/100,000 in 2020 (Fig. 3A). However, the incidence showed higher increased times from 2017 to 2020 (3.5-fold, 76.7 cases per 100 000 person-years to 265.9 cases per 100 000 persons-years) than from 2009 to 2017 (1.3-fold; 59.8 cases per 100 000 person-years to 76.7 cases per 100 000 persons-years). A shift of the varicella cases to older age groups was observed, with the peak proportion of cases shifting from 5–6 years old to 7–8 years old, which increased from 14.3% in 2009–2011 to 26.0% in 2018–2020 (Fig. 3B and C).

#### Vaccine effectiveness of varicella

**Fig. 4:**
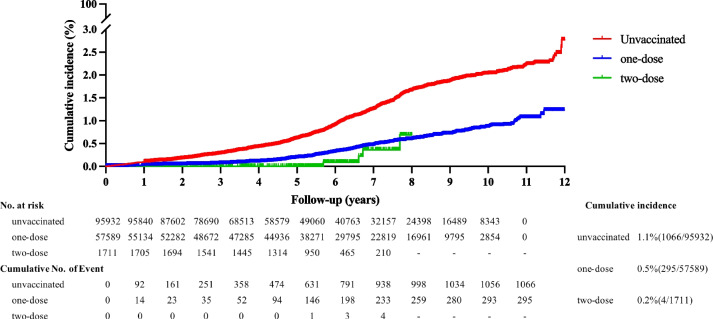
The cumulative incidence of varicella infection

A total of 1,365 cases were successfully linked between the NNDRS and IIS and were included in the analysis of VE of varicella. The incidence densities of the unvaccinated and one-dose were 163.0 and 72.6 per 100,000 person-years, respectively (Table 3). The cumulative incidence of the unvaccinated and one-dose groups was 1.1% and 0.5% (Fig. 4).

**Table 2:** The HR and confounding variables for varicella infection

Variable	HR (95% CI)	*P* value
one-dose	0.405(0.356,0.461)	< 0.0001
Age at entry into cohort	0.583(0.5,0.681)	< 0.0001
Year of birth	1.337(1.299,1.376)	<0.0001
Immunization management institution	0.987(0.979,0.995)	0.0016

The variables of age at entry into the cohort, year of birth and immunization management institution (a total of 24 township health centers) were significant confounders noted in the univariate model and were adjusted for in the multivariate Cox regression model

As shown in Table 2, year of birth, age at cohort entry and immunization management institution were significantly associated with VE, which were all adjusted as covariates in the Cox regression model. The overall adjusted effectiveness of one dose of varicella vaccine was 56.5% (Table 3). The effectiveness of the one-dose varicella vaccine decreased steadily with the follow-up time. The adjusted VE of the one-dose group was 72.9% during the initial 4 year and decreased to 41.8% during years 7 to 12 (Table 4).

**Table 3:** Estimated VE against varicella in different vaccination statuses

Vaccination status	No. of cases	Follow-up time, person-years	The incidence density, per 100,000 person-year	VE		
				Crude VE	Unadjusted VE	Adjusted VE
Unvaccinated	1066	653,794.3	163.0	Ref	Ref	Ref
one-dose	295	406,527.3	72.6	((49.4%,60.9%)	59.5%(53.9%,64.4%)	56.5%(48.9%,62.9%)

The crude IRR was calculated by dividing the incidence in the one-dose by the incidence in the unvaccinated group. The unadjusted HR was estimated using a Cox regression model with cases as a response variable and vaccination status. The adjusted HR was estimated using a Cox regression model with cases as a response variable and vaccination status, year of birth, age at cohort entry, and immunization management institution as explanatory variables. *VE* Vaccine effectiveness. VE = 1-HR. *IRR* Incidence rate ratio, *HR* Hazard ratios

**Table 4:** Estimated VE against varicella in different vaccination statuses stratified by follow-up time

Stratification variable	Vaccination status	No. of cases	Follow-up time, person-years	The incidence density, per 100,000 person-year	VE		
					Crude VE	Unadjusted VE	Adjusted VE
[0–4)	Unvaccinated	358	390,017.7	91.8	Ref	Ref	Ref
	one-dose	52	213,295.5	24.4	73.4%(64.6%,80.1%	74.3%(65.6%,80.8%)	72.9%(61.0%,81.1%)
[4–7)	Unvaccinated	433	163,124.1	265.4	Ref	Ref	Ref
	one-dose	146	124,581.1	117.2	55.8%(46.7%,63.4%)	56.3%(47.3%,63.8%)	56.8%(45.6%,65.8%)
[7,12)	Unvaccinated	275	100,652.5	273.2	Ref	Ref	Ref
	one-dose	97	68,650.6	141.3	48.3%(34.8%,59.0%	48.5%(35.0%,59.2%)	41.8%(22.4%,56.4%)

The crude IRR was calculated by dividing the incidence in the one-dose by the incidence in the unvaccinated group. The unadjusted HR was estimated using a Cox regression model with cases as a response variable and vaccination status. The adjusted HR was estimated using a Cox regression model with cases as a response variable and vaccination status, year of birth, age at cohort entry, and immunization management institution as explanatory variables. *VE* Vaccine effectiveness. VE = 1-HR. *IRR* Incidence rate ratio, *HR* Hazard ratios

## Discussion

To our knowledge, this is the first study to describe varicella epidemiology and the long-term vaccine effectiveness under vaccination coverage less than 50%. In this register-based birth cohort study, there was an increasing trend of annual incidence of varicella infection under vaccination coverage less than 50%. A shift of the varicella cases to the older age groups was observed, with the peak proportion of cases shifting from 5–6 years old to 7–8 years old. The adjusted effectiveness of one dose of vaccine waned over time.

Several factors, such as demographic, etiological and insufficient vaccine coverage could contribute to the appearance of growing incidence [19]. In countries with varicella vaccination programs (vaccine coverage over 80%), there was a 57% ~ 97% reduction in the incidence of varicella cases after the introduction of a one- or two-dose vaccine [20, 21]. Frustratingly, in our study, the 1-dose vaccination coverage was 43.1%-49.4% in the 2008–2013 birth cohort and then decreased to 20.1%-32.0% in the 2014–2018 birth cohort. Correspondingly, the annual incidence of varicella infection increased 4.4-fold from 59.8/100,000 in 2009 to 265.9/100,000 in 2020. The increased times from 2017 to 2020 (3.5-fold) was higher than the increase from 2009 to 2017 (1.3-fold). The significant increase in incidence since 2017 may due to a further decrease in vaccination coverage and an increased sensitivity of varicella surveillance since varicella designation as a Class “C” infectious disease in Jiangsu Province, as we described in methods. The finding of this cohort study was consistent with a previous mathematical modeling study which revealed that if one-dose vaccination coverage remains moderate (30% ~ 70%) for a long time, there is a high risk of an increase in incidence and mortality [1, 22]. Another mathematical model study showed that coverage of varicella vaccination was a critical factor in significantly reducing the number of varicella cases, with a 20% to 30% reduction in varicella cases when the vaccine coverage was increased by 20% [23]. In addition, another study showed that even moderate coverage with two doses of varicella vaccination may lead to an increase in adult varicella cases [24]. A eleven-year community-based cohort study from China found the incidence of varicella increased annually under the overall vaccination coverage of 71.7% [25], which is similar to our study. Another study also shown an increase in varicella incidence from 2007 to 2011 with the one-dose coverage between 70 to 80%, and an significant decrease in incidence since one-dose vaccination coverage increase to 90% and the implementation of an two-dose vaccination program [26]. Furthermore, mathematical model study also showed that moderate (30% ~ 70%) one-dose vaccination coverage can lead to an upward shift in the age distribution of varicella [1, 22]. In this study, we also observed a shift in varicella peaks from 5–6 years old to 7–8 years old. Therefore, it is urgent to increase the varicella vaccine coverage to 80% to reduce the incidence of varicella and prevent any potential shift in the age at infection in China.

In our study, the overall adjusted effectiveness of one dose of varicella vaccine was 56.5%. One meta-analysis estimated the global varicella VE is 81% (95% CI: 78%–84%) for one dose and 92% (95% CI: 88%–95%) for two doses [10], which is higher than our studies. This may be due to the effect of herd immunity, as the majority of studies included in the meta-analysis were from countries with universal varicella vaccination. One 8-year community-based cohort study from China reported that the one- and two-dose varicella VE was 50.3% and 98.7% [27], respectively, which was similar to our study. The 1-dose varicella vaccine is insufficient to prevent varicella outbreaks even in areas of high coverage [28–30]. Hence, the recommendation for a two-dose varicella vaccine has been proposed, and universal vaccination policies have been introduced in many countries, including the United States, Canada, Japan, Germany, and Luxembourg [1]. Several studies demonstrated that a two-dose universal vaccination regimen of varicella vaccine significantly reduced the incidence of varicella [26, 31–33]. Until 2023, the strategy for varicella vaccination in Jiangsu is to encourage people to receive vaccine voluntarily and at their own expense, which is in line with the national vaccine in China. In order to reduce the incidence of varicella, Jiangsu government decide to introduce varicella vaccination in the routine childhood immunization programme with two-dose schedule since Jan 1, 2023. Our findings suggest that the CDC in Jiangsu province should make great effort to ensure reaching one-dose vaccine coverage over 80%, and implement two-dose catch-up vaccination for children as soon as possible. We will continuous monitoring varicella vaccine impact on varicella incidence after routine two-dose varicella vaccination program in the Jiangsu province.

The duration of protection after one dose is still unclear. A 14-year prospective study reported no reduction in VE over time [34], while more studies reported decreases in VE over time. A community-based study reported that one-dose VE decreased from 73.1% of the 2013 birth cohort to 26.8% of the 2008 birth cohort; two-dose VE decreased from 99.3% of the 2013 birth cohort to 93.9% of the 2008 birth cohort [27]. In our study, the adjusted VE decreased from 72.9% to 41.8% in the one-dose group after 12 years. In addition, our study found that VE is stable in the first 1 to 4 follow-up years, but decreases significantly after 5 follow-up years in our study, indicating that varicella vaccine-induced immunity appears to wane over time [35]. Similar findings have been reported in the United States and Turkey, which reported that the vaccine recipients were more than twice or three times as likely to develop moderate/severe breakthrough ≥ 5 years after vaccination compared to those who had been vaccinated more recently [35, 36].

The following limitations existed in this study. First, NNDSS is a passive surveillance system with a certain rate of missed reports. Therefore, the incidence of varicella might have been underestimated. Second, the majority of varicella cases in this study were based on clinical definitions; therefore, there may be clinical diagnostic omissions, especially in vaccinated patients with mild varicella rash symptoms. Finally, the small number of two-dose recipients might limit the statistical inference in the two-dose VE, and therefore the two dose group was excluded from the VE estimates. Nevertheless, this study, which was based on community surveillance, is still fairly representative of real-world data in Ganyu County, Jiangsu Province, China.

In conclusion, this study found that the annual incidence of varicella infection increased, and a shift of the varicella cases to older age groups occurred, which may be may be attributed in part to the insufficient vaccination coverage. The effectiveness of one dose of varicella vaccine was moderate and waned over time. It is urgent to increase the varicella vaccine coverage to 80% to reduce the incidence of varicella and prevent any potential shift in the age at infection in China.

## Data Availability

The study data are available for academic purposes upon reasonable request. To gain access, please contact corresponding author Ying-ying Su for further details.

## Abbreviations

DFA: Direct immunofluorescence antibody assay
HR: Hazard ratio
IIS: Immunization information system
IRR: Incidence rate ratio
NNDSS: National Notifiable Disease Surveillance System
PCR: Polymerase chain reaction
SE: Standard error
VE: Vaccine effectiveness
WHO: World Health Organization
